# Cortical Plasticity Induced by Short-Term Multimodal Musical Rhythm Training

**DOI:** 10.1371/journal.pone.0021493

**Published:** 2011-06-29

**Authors:** Claudia Lappe, Laurel J. Trainor, Sibylle C. Herholz, Christo Pantev

**Affiliations:** 1 Institute for Biomagnetism and Biosignalanalysis, University of Muenster, Münster, Germany; 2 Department of Psychology, Neuroscience & Behaviour and the McMaster Institute for Music and the Mind, McMaster University, Hamilton, Canada; 3 Montreal Neurological Institute, McGill University, Montreal, Canada; University of Southern California, United States of America

## Abstract

Performing music is a multimodal experience involving the visual, auditory, and somatosensory modalities as well as the motor system. Therefore, musical training is an excellent model to study multimodal brain plasticity. Indeed, we have previously shown that short-term piano practice increase the magnetoencephalographic (MEG) response to melodic material in novice players. Here we investigate the impact of piano training using a rhythmic-focused exercise on responses to rhythmic musical material. Musical training with non musicians was conducted over a period of two weeks. One group (sensorimotor-auditory, SA) learned to play a piano sequence with a distinct musical rhythm, another group (auditory, A) listened to, and evaluated the rhythmic accuracy of the performances of the SA-group. Training-induced cortical plasticity was evaluated using MEG, comparing the mismatch negativity (MMN) in response to occasional rhythmic deviants in a repeating rhythm pattern before and after training. The SA-group showed a significantly greater enlargement of MMN and P2 to deviants after training compared to the A- group. The training-induced increase of the rhythm MMN was bilaterally expressed in contrast to our previous finding where the MMN for deviants in the pitch domain showed a larger right than left increase. The results indicate that when auditory experience is strictly controlled during training, involvement of the sensorimotor system and perhaps increased attentional recources that are needed in producing rhythms lead to more robust plastic changes in the auditory cortex compared to when rhythms are simply attended to in the auditory domain in the absence of motor production.

## Introduction

Musicians and non musicians exhibit structural and functional differences in a wide range of brain areas [1]–[12]. They show increased cortical representations for tones of the musical scale [1], [13], [14], [15], for chord sequences [16] and melodies [17]–[18], even when melodic predictions are generated by imagery [19].

Enjoyment of music relates to familiarity with musical genres that help the listener to form and develop perceptual expectations for musical events. In that sense pitch, harmony, timbre and rhythm establish a musical predictive template that produces musical expectations [20]. Violations of those expectations are reflected in an electrophysiologically measurable event related response, the mismatch negativity (MMN).

Musical pitch expectations can be quickly formed by short-term musical piano training that shapes the brain activation within the auditory cortex [21]. After eight sessions of multimodal piano training in the form of learning to play short melodic chord sequences on a keyboard, non musicians showed an increased MMN in response to pitch incongruence, especially in the right hemisphere. A control group, that merely listened carefully to and made judgements about the music played by the experimental group, showed no MMN enhancement. Thus, the multimodal integration, the co-activation of auditory and sensorimotor areas and attentional mechanisms, that are involved in musical training, likely contribute to the brain plasticity effects that have been shown in musicians.

In the same way that the chord structure of a musical piece shapes expectations about upcoming melodic events, the temporal structure of a musical piece induces anticipation of rhythmic events in the listener. A number of studies indicate that interactions between the auditory and motor systems may be particularly strong when rhythm is involved [22]–[30]. We therefore hypothesized that musical training, that is focused on the rhythm of a melody, should lead to enhancements of rhythm perception and correspondingly to enhancements of the cortical responses to deviations in rhythmic musical material. Since several studies suggest that the left hemisphere is specialized for temporal processing [31], [32], [33], we hypothesized further that neural activation increases induced by the rhythmic training should be particularly pronounced in the left hemisphere. Thus, the goal of this study was to investigate how rhythmic expectations within a musical context can be changed by short-term musical training involving either listening or learning to play the piano in a highly controlled laboratory environment.

## Methods

To investigate this hypothesis we measured the MMN to rhythmic deviants before and after sensorimotor-auditory or auditory musical training. Musical training strengthens expectations for musical events, which is reflected in the auditory system as better performance on discrimination of tonal frequencies [34] and temporal events [35]. This ability can be quantified electrophysiologically in humans by means of completely non-invasive electro- or magneto-encephalographic measurements of the MMN (MMN in EEG, MMNm in MEG). MMN is a pre-attentive fronto-central negative component of the event related field, measured at latencies between 120 and 250 ms after stimulus onset with brain sources within the primary and secondary auditory cortices [21], [36]. The MMN component can be elicited by changes in auditory features such as frequency, intensity or duration of a sound, but it can also reflect violations of more complex aspects of auditory features [17], [37]. In the present study the duration mismatch negativity was used to determine changes in cortical strength after a rhythmic incongruency.

### Subjects

Twenty-four non musicians (14 females) between 24 and 38 years of age participated in the study. Participants had no formal musical training, except for their compulsory music lessons at school. The data of four subjects had to be excluded because of the very low signal-to-noise ratio (insufficiently pronounced MMN). Thus 20 subjects were included in the analyses. Subjects were all right-handed as assessed by the Edinburgh Handedness Inventory [38]. None of the subjects had a history of otological or neurological disorders. We used pure tone audiometry to confirm normal audiological status. Subjects were informed about the nature of the study, which was approved by the Research Ethics Board of the University of Münster. Based on a clear understanding of what participation involved, subjects gave informed consent to take part in this study. Subjects were randomly assigned to the different experimental goups (sensorimotor-auditory, SA and auditory, A). The SA-group learned to play a musical sequence on the piano, whereas the A-group merely listened carefully to the music that was played by the participants of the SA-group and evaluated whether the sequences were rhythmically correct or not.

### Stimuli

**Figure 1:**
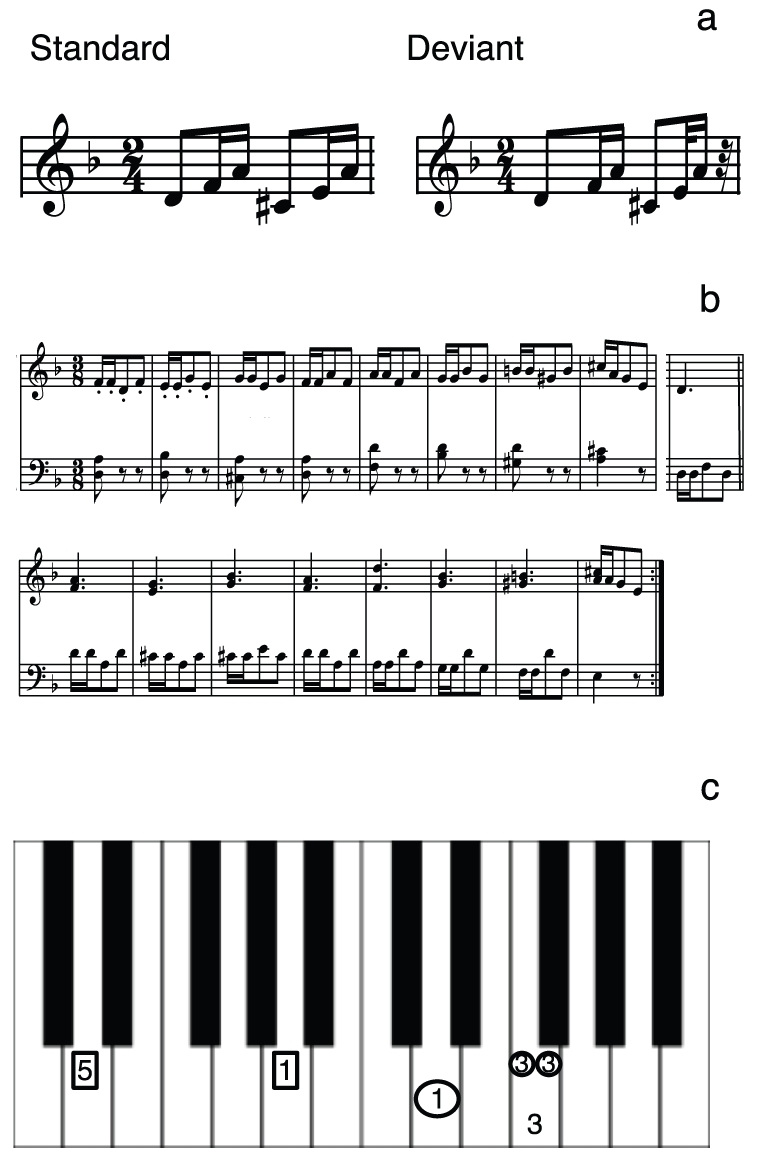
Stimulus material. (a) Tone sequences for the standard and the deviant stimuli that were used in the MEG measurements before and after training. (b) Musical score of the training piano melody. The first line is repeated after the end of the second line. The measure at the end of the first line concludes the melody. (c) Visual templates for the SA training. Numbers represent the fingers (thumb, 1; index finger, 2 and so on) with which the subjects were supposed to press the corresponding piano keys. The rectangles indicate that the left hand is used, the circles mark the right hand. The numbers that were depicted in one horizontal line had to be played simultaneously. The small circles indicated that notes had to be played at double speed.

The musical stimuli for the MEG measurement before and after training comprised six-tone piano sequences generated in a realistic piano timbre with a digital audio workstation (Figure 1). The sequences were composed of a d-minor broken chord in root position followed by an A-major chord in first inversion: d' (293.66 Hz) - f' (349.23 Hz) - a' (440.00 Hz) - c sharp (277.18 Hz) - e' (329.63 Hz) - a' (440.00 Hz). These are the two most important chords (tonic and dominant) in the key of d-minor, the key of the training exercises described below. The standard stimulus was composed of two rhythmic figures, each with an eighth note (400 ms) at the beginning followed by two sixteenth notes (200 ms each) for a total duration of 1600 ms. The deviant stimulus (c.f. Figure 1a), was identical to the standard except that the fifth tone was shortened by 100 ms to produce a duration advance deviance of 100 ms on the sixth note and a total sequence duration of 1500 ms. The two sequences (standard and deviant) were presented in an oddball paradigm with two runs of 400 trials separated by a short break. Each run consisted of 320 standards and 80 deviants presented in a quasi random order such that at least three standards occurred between two deviants. Note that the rhythmic motive used during the MEG measurement was not identical to that used during training so that we could test the training effects under conditions requiring some generalizability. Specifically, the order of the long and short notes was reversed. Our previous melody study showed that participants were able to abstract harmonic rules from training material and to transfer them to new musical material. Thus, a similar effect in the present study would allow us to draw conclusions about generalization of training effects in the rhythmic domain as well. Although Western music offers a large variety of melodic and also rhythmic material, it is nevertheless based on a comparatively small rule catalogue, and it lies in the nature of musical training that it generalizes to different musical material. We wanted to demonstrate the potentials of musical training for musical learning in general.

### Training procedure

The first 16 measures of an exercise from a piano workbook for beginners [39] were used for the piano training (Figure 1b). In order to avoid possible differential plasticity effects in the two hemispheres due to dissimilar movements of the two hands, we chose a piano piece where both hands were similarly involved. The piano exercise was in d-minor with a metrical time signature of 3/8. The melody was built from a recurring small rhythmic motive consisting of two sixteenth notes on the first beat followed by two eighth notes on the second and third beats. The rhythmic motive did not change during the whole piece. In the first 8 measures the melody was in the right hand whereas in the last 8 measures the left hand played the melodic line. In each case the other hand played an interval on the first beat of each measure. During the first 8 bars the motive was played on successively higher scale steps each bar, and during the final 8 bars on successively lower scale step each bar. In order to facilitate training, we did not use the musical notation of the piano exercise, but visual templates instead (Figure 1c). On each template the image of the piano keyboard was depicted and the finger placement was marked.

The SA-group was instructed in how to play the piano exercise. The piano sequence was demonstrated by the experimenter at the beginning of the first training session. Training sessions were scheduled on 8 days within two weeks, each session lasting 30 minutes. A computer recorded the keystrokes of the subjects during the training through a MIDI connection. The MIDI data of the SA-group provided the stimuli for the training of the A-group. This included also the first training sessions when piano performance of the SA-group was still poor. Consequently, musical exposure of the SA- and A-group was identical. Each participant of the A-group was paired with a participant of the SA-group and listened to all the training session of that subject. Prior to training the A-group received a short introduction to the correct piano exercise. As in the SA-group, training sessions for the A-group were scheduled on 8 days within two weeks. During auditory training subjects of the A-group were seated in front of the piano. However, they received no visual information regarding the keys that had been pressed. Subjects in the auditory group were instructed to press the right foot-pedal whenever they noticed that the rhythm was played incorrect. This task ensured that subjects of the auditory group listened thoroughly and participated actively in the experiment.

### Behavioral test

To evaluate the effect of the training on a behavioral level, all subjects participated in an auditory discrimination test before and after the two-weeks of training. For this test we extracted the first two measures of the piano exercise and recorded them via MIDI connection to a computer. Thus we obtained a sequence that contained 8 notes in the melody part and two accompanying intervals on the first beat of each measure. During the behavioral test this sequence or temporally altered sequences were presented. In temporally altered sequences a randomly chosen note of the melody was played earlier or later than expected by 10, 20, 30, 40 or 50 ms. These temporal offsets were chosen through pilot testing, which had revealed that a 50 ms time shift is easy to detect even for non musicians, whereas a 10 ms or even 20 ms time shift is very hard to detect. Sequences with temporal errors were presented randomly interleaved with correct sequences and subjects responded by pressing the left-foot pedal of the piano whenever they detected a temporal advance or delay of a note. If they did not detect a temporal error they had to press the right foot-pedal to start the next trial. The test contained 243 trials and lasted about 20 minutes. The first three trials were correct sequences to accustom the subject to the task. The remaining 240 trials contained 120 error sequences and 120 correct sequences. Each time shift was thus repeated 12 times during the test.

### MEG data acquisition

The auditory MMN responses were measured from all participants before and after training. Training induced plasticity was evaluated by comparing the MMN differences before and after training between the SA and A-groups.

Magnetic field responses were recorded with a 275-channel whole-cortex magnetometer system (Omega 275, CTF Systems). The MEG signals were low-pass filtered at 150 Hz and sampled at a rate of 600 Hz. For each individual subject, epochs of 2 s for the standard and of 1.9 s for the deviant stimulus beginning 0.2 s before the last tone of the stimulus and ending 0.4 s after stimulus offset were extracted from the continous data set. The total recording time was 35 min. The recordings were performed in a magnetically shielded and acoustically silent room. The subjects were in an upright position, seated as comfortably as possible while ensuring that they did not move during the measurement. Three localization coils, that were fixed to the nasion and the entrances of both ear canals, were used to check the subject's head position at the beginning and end of each recording block. Subjects were instructed to move and blink as little as possible, to stay relaxed but awake during the measurement, and to pay no attention to the sound stimuli. Alertness and compliance were verified by video monitoring. To control for confounding changes in attention and vigilance, subjects watched a soundless movie of their choice, which was projected on a screen placed in front of them.

### MEG data analysis

The recorded magnetic field data were averaged separately for the standard and the deviant stimuli. Epochs contaminated by muscle or eye blink artifacts containing field amplitudes greater than 3 pT in any MEG channel were automatically rejected by the averaging procedure. The MMN was expected to be elicited in the deviant sequences after the onset of the sixth tone. Therefore, standard and deviant datasets were temporally aligned to the onset of the sixth tone of the sequence and subtracted to generate difference waveform data sets, representing the MMN.

Although the alignment ensures that the onset time and duration of the sixth tone of the standard and the deviant were identical in both sequences there is still the possibility that the fifth tone, which is shorter in the deviant than in the standard sequence, provides additional MEG components that interfere with the subtraction procedure. Since the fifth tone of the deviant sequence is of shorter duration than the standard one, its corresponding N1 response will be closer to the onset of the sixth tone and this N1 component could then be mistakenly interpreted in the deviant-standard difference waveform as an MMN component [37]. In two test measurements with four musically experienced subjects we therefore tested a different subtraction procedure. The two stimuli were presented in two blocks as standard and deviant as described above. Then, in two further measurement blocks the roles of standard and deviant were reversed, i.e., the standard became the deviant and vice versa. This procedure enabled subtraction of physically identical stimuli, namely the shorter stimulus that was the standard in the latter measurement from the identical shorter stimulus that was the deviant in the earlier blocks. We compared the results of this subtraction procedure with that of the direct subtraction procedure in which the shorter deviant and the aligned longer standard were subtracted. Both subtraction methods yielded the same results, that is, the obtained MMN components were nearly identical. Since the direct subtraction method required a much smaller number of trials the direct subtraction procedure was employed in the main experiment.

For the MMN source analysis a baseline correction was performed relying on the 100 ms time interval prior to the onset of the piano tone sequences. Then, the source analysis model of two equivalent current dipoles (ECD) (one in each hemisphere) was applied to the MMN component identified in the data between 120 to 180 ms after tone onset. The two spatiotemporal dipoles, defined by their dipole moment, orientation, and spatial coordinates, were fitted simultaneously to the MMN derived from the difference waveforms for both hemispheres and for each recorded dataset before and after training. The source space projection method [21], [40] was applied, collapsing the 275 channel data to one source waveform for each dipole. Finally, grand average waveforms for the dipole moments were computed for pretraining and posttraining data, groups (SA and A), and hemisphere (left and right). To evaluate the MMN source strength across participants, the MMN dipole moment peaks were determined from the corresponding waveforms of each individual participant and subjected to statistical analysis by means of a repeated measures mixed-model ANOVA with factors group, pretraining/posttraining and hemisphere. In all statistical tests, the alpha-level was set at 0.05, and all test were two-tailed unless otherwise stated.

## Results

### Training

Prior to the training procedure participants of both groups listened to a correct version of the piano exercise. The subjects of the SA-group started in their first training session to play the upper line of the piece with the right hand only. The left hand was added in later training sessions after subjects were able to play the first part with the right hand correctly. After finishing the first line of the piano piece the same procedure was applied for the second line. The employment of the left and right hand was reversed, however, since the melody in the second part of the piece was in the left hand. The transfer of the melody to the left hand was difficult for most of the subjects, but was eventually mastered by all participants. Due to differences in learning progress among subjects, the different steps during the training process (inclusion of the second hand, switching to the second line) were performed at different times during the training of each individual subject. At the end of the training, after 8 sessions, all subjects of the SA-group were able to play the piece within an acceptable speed with few mistakes. However, two subjects only reached successful performance of the second line with a reduced accompaniment in the right hand. Instead of playing the complete intervals, they simply played a single accompanying tone in each measure.

### Behavioral test data

The Performance on the behavioral test was evaluated by computing the detection rate for the error trials of each absolute time shift (10, 20, 30, 40, or 50 ms). Positive and negative time shifts were analyzed together. The data from one subject in the A-group (due to technical failure) and two subjects from the SA-group (due to misunderstanding the task) had to be excluded, so that overall the data of 9 subjects of the A-group and 8 subjects of the SA-group were analyzed.

**Figure 2:**
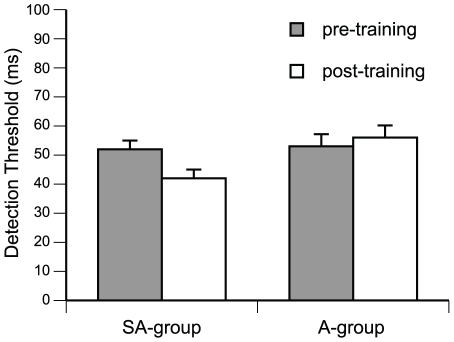
Group means of behavioral performance in the auditory discrimination test before and after training: pre, pretraining; post, postraining. Error bars indicate SEM.

The detection rates were fitted with a Weibull function and the 75% detection threshold was determined. A 2×2 mixed model ANOVA with factors group and pretraining/posttraining revealed a significant interaction of group x pretraining/postraining, (F (1,17) = 5.098; p = 0.039), demonstrating that rhythmic discrimination ability improved more strongly in the SA than in the A-group. On average, the detection threshold in the SA-group improved by 9 ms (Figure 2). No threshold improvement was observed in the A-group. Main effects of group and session did not reach statistical significance.

### MEG data

**Figure 3:**
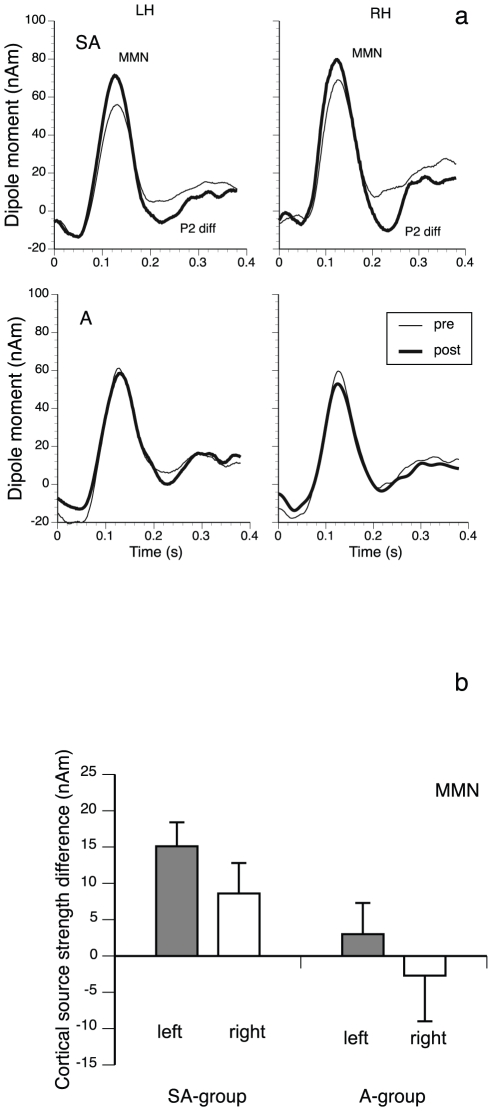
MMN results. (a) Group averages of the source waveforms obtained after performing source-space projection before and after training for both groups and hemispheres. Data for the SA-group are shown in the upper row, data for the A-group are shown in the bottom row. Data from the left hemisphere (LH) are presented on the left and those of the right hemisphere (RH) are presented on the right. Thin lines indicate pretraining and thick lines posttraining data. (b) Group averages of the pretraining/posttraining differences of the individual MMN source waveform peak amplitudes from both groups and hemispheres. Left: left hemisphere; right: right hemisphere; seq: sequence. Error bars indicate SEM.

The MEG data showed a clear MMN dipolar pattern in most of the individual subjects, which justified the use of a single equivalent current dipole model for the cortical source analysis of the data. Figure 3a shows the averaged source waveforms obtained after the performed source space projection for the pre- and posttraining conditions in both groups. A clear MMN is discernible in all panels. The MMN is similar in size in both groups in the pretraining condition. A well-pronounced enhancement of this component in the posttraining condition is visible in the SA-group. In contrast, in the A-group no clear MMN change was observed.

Group averages of the pre/post-training differences of the individual MMN source strength peak amplitudes are shown in the bar plots of Figure 3b. A mixed model ANOVA with factors group, pre-training/post-training and hemisphere gave a significant main effect of training, (F (1,18) = 6.54; p = 0.022), indicating that there was an overall training effect for both groups, and a pre/posttraining x group interaction, (F (1,18) = 4.83; p = 0.044), indicating that the training effect in the SA-group was stronger than in the A-group. The three-way interaction pre/posttraining x group x hemisphere was not significant, F(1,18) = 0.025; p = 0.88.

**Figure 4:**
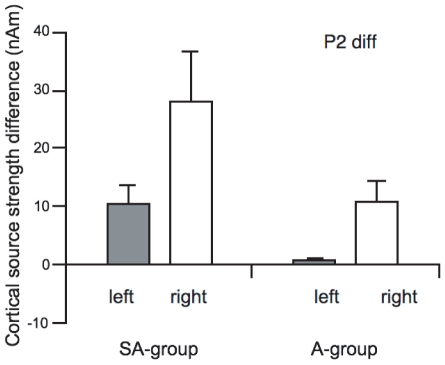
Group averages of the pretraining/posttraining differences of the individual P2 source waveform peak amplitudes from both groups and hemispheres. Left: left hemisphere; right: right hemisphere; seq: sequence. Error bars indicate SEM.

A further observation was a difference in source strength between pre- and post training in the SA-group at about 230 ms. This indicates an increase in the P2 wave of the slow auditory evoked response to the deviant stimuli after training. The group averages of the pre-training/post-training differences of the individual source strength amplitudes of the P2 difference between deviant and standard were therefore tested for statistical significance (Figure 4). A mixed model ANOVA revealed a significant main effect of training, (F(1,18) = 13.69; p = 0.002), and a significant main effect of hemisphere, F(1,18) = 8.76; p = 0.009, indicating an overall training effect in both groups and an overall stronger P2 response in the right hemisphere. The ANOVA further revealed a significant interaction between training and group, (F(1,18) = 13.52; p = 0.002), showing that the P2 training enhancement was especially pronounced in the SA-group. The three way interaction training X hemisphere X group, (F(1,18) = 4.46; p = 0.051), indicated that the P2 training enhancement of the SA-group was particularly strong in the right hemisphere.

## Discussion

Rhythm-focussed sensorimotor-auditory training in non musicians results in representational changes in the auditory cortices. The SA-group that had received sensorimotor-auditory piano training showed a significant post-training enhancement of the MMN to temporal deviants in rhythmic sequences. The A-group that had received only auditory training showed no significant training effect on MMN. This is consistent with the behavioral finding that thresholds for detecting temporal errors only improved in the SA-group but not in the A-group. However, both groups showed significant enhancement of the P2 component between deviant and standard after training, although the enhancement was larger in the SA-group, indicating that even the auditory-only training led to some plastic changes in auditory cortex. Previous studies indicate that the P2 component is larger in skilled musicians [14] and is highly neuroplastic with frequency discrimination training [41], [42]. Our data extend these findings by showing that the P2 difference component, which is mainly based on the training effect on the P2 response to the deviants, is also sensitive to rhythmic processing after short-term training. In sum, in comparison to the A-group, the SA-group showed significantly larger training effects on behavioural thresholds, MMN amplitude, and P2 amplitude.

Training and test stimuli were not identical, indicating that the multimodal effects of the training generalized. Because MMN and P2 are generated primarily in auditory cortices [43], the results point to strong effects of sensorimotor practice on auditory representations for musical rhythm. These results extend the findings of our previous study which described increased neural activation within the auditory cortex in the form of MMN enhancement after musical training that focussed on melodic chord progressions [21]. Whereas the present study examined brain responses to deviants in the temporal domain, the previous study examined responses to deviants in the pitch domain. Perhaps most interesting is that for both pitch-based and rhythm-based training, larger plastic enhancements of responses from auditory cortex were seen after sensorimotor-auditory training than after auditory-alone training, suggesting that multimodal stimulation has a larger effect on auditory cortex than auditory stimulation alone.

Several studies show different brain responses in musicians and non musicians to pitch-, melodic- or harmonic-based deviants [16], [17], [18], [19], [21] or during rhythm perception [23], [32], [44], [45], [46], [47]. However, when comparing adult musicians and non musicians, it is difficult to determine definitively whether the differences seen are primarily the result of the extensive experience of the musicians in practicing their instruments or whether they are largely a result of pre-existing congenital differences that led to the decision to undertake extensive musical training. The design of our study exposes the effects of experience directly. Because we randomly assigned subjects to different training groups, and because we controlled the experience and measured responses before and after training, we can conclude that the effects that we report are the result of the experience itself.

Our finding of superior learning with multimodal training is in line with other evidence that the brain is very sensitive to relations across modalities. The interaction and integration of different sensory modalities is especially important when playing a musical instrument. The multimodal effects that we obversed likely involved both somatosensory and motor interactions with auditory processing. Previously, Schulz et al. [48] found evidence for auditory/somatosensory reorganization of cortical functions in musicians by comparing trumpet players and control subjects who had never played an instrument. In the trumpet players, concurrent stimulation of the lips and presentation of a trumpet tone led to a stronger cortical activation compared to the sum of the responses to the two types of uni-modal stimulations, either trumpet tone or tactile lip stimulation. In the present study, it is likely that in the SA- group, the concurrent experiences of touching the keys (somatosensory) and hearing the piano tones (auditory) led in part to the enhanced learning seen in this group.

As far as the importance of the motor aspect of our training protocol, the concept of a strong link between the auditory and motor systems has a long history [49]. Musical stimuli give rise to rhythmical organized motor behavior [27], [28], [30], and synchronized movement to music is found in all cultures [50]. Even 5- to 25-months-old infants coordinate their movement to musical rhythmic stimuli and adapt the tempo of their rhythmic movement to the tempo of the auditory rhythmic stimuli [51]. Executing rhythmic movements involves a network of brain areas spanning the basal ganglia, cerebellum, motor, premotor cortex, and supplementary motor cortex [30]. Recent fMRI studies have shown, however, that these movement-related areas are also activated during auditory perceptual tasks [24], [52]. In particular, the cerebellum [53]) and the premotor cortex [22] are activated during auditory discrimination, and disruption of auditory feedback affects motor execution [54].

In addition, the results of the present study indicate that the interaction between auditory and motor areas is bidirectional, suggesting that movement can affect auditory processing. Phillips-Silver & Trainor [25], [26] showed that for both infants and adults, bouncing on every second beat of an auditory metrically-ambiguous rhythm pattern biased listeners to hear the ambiguous pattern as a march whereas bouncing on every third beat of the same pattern biased them to hear the same ambiguous pattern as a waltz. Recent physiological evidence also indicates strong bidirectional connections between auditory and movement-related areas [30]. For example, auditory cortex is activated when musicians observe someone else play a keyboard [55]. Furthermore, similar auditory and motor areas are activated when pianists play a piece without being able to hear it and when they listen to it without playing it [56], [57]. The results of the present paper are consistent with all of these findings, demonstrating that sensory-motor training affects auditory cortical areas.

Since the mismatch negativity is mainly generated in the auditory cortex, the mismatch paradigm is not suited to directly investigate response changes in motor related areas to musical stimuli after musical training. A different experimental design would be needed to demonstrate directly this connection. However, we suggest that auditory-motor interaction is bidirectional because the auditory input was identical for both groups, the only difference was motor-execution with associated attentional mechanisms in the SA- group. We therefore reason that auditory-motor interactions are likely involved in the generation of the increased mismatch negativity after sensorimotor training.

Whereas many studies show larger MMN in the right compared to left hemisphere, the present results showed no difference in hemispheric involvement. It is possible that MMN tends to be right-hemiphere dominant for pitch-based discriminations, but not for duration-based discrminations. Indeed, our previous training study involving melodic chord sequences showed a greater plasticity effect in the right hemisphere whereas the present rhythm training study showed plastic changes of similar magnitude in both hemispheres. The strong involvement of the right hemispheric auditory cortex in the melody study, and the relatively well-pronounced involvement of the left auditory cortex in the rhythm study are consistent with data showing preferential encoding of spectral information on the right and temporal encoding on the left [31], [32], [58], [59], [60].

It has also been suggested that musical expertise could lead to a higher degree of analytical processing, which is believed to favor left hemispheric mechanisms [61], [62]. The results from our study are somewhat more complicated in that we found statisically equivalent effects of training in the right and left hemispheres in the SA-group for MMN, but significantly greater effects of training in the right than left hemisphere for the P2 component in both the SA and A-groups. One reason for our findings might be that our stimuli included also pitch and melodic variation, which might have interacted with the rhythmic elements [63]. Another reason might be that the sensorimotor-auditory training was too short to reveal differential hemisphere effects.

Playing the piano is a motivating and demanding task. It is thus conceivable that the participants in the SA-group were more motivated or more engaged in their task than the participants of the A-group. The stronger MMN plasticity in the SA-group, therefore, might originate also from a stronger involvement of motivation or attention in this group. Attention, and other top-down modulatory signals, can increase plasticity effects in the auditory cortex [64], [65], [66]. However, the participants of the A-group also had to concentrate on a task that demanded alertness and attention, namely, the detection of the rhythmic errors in the auditory material of the SA-group. Thus, their attention was directed to the same stimulus feature (rhythmic correctness) as in the SA-group, such that the level of attention on the auditory input was likely comparable in both groups. The groups differed in that the SA group performed motor behavior and acted to create the acoustic material, while the A group merely listened attentively to the created material. Thus, while we cannot rule out that attentional or motivational factors differed between the groups, any difference in that regard would be driven by the active involvement and the motor behavior of the piano playing.

We conclude that sensorimotor-auditory training of rhythmic material can increase the neural responses to a temporal mismatch in non musicians. The response increase was achieved after only eight training sessions. The enhancement of cortical activity was based on new musical material, suggesting strong generalization effects of sensorimotor-auditory training. Since cortical activity was enhanced to a lesser degree in the control group after auditory only training, we conclude that rhythm-focussed multimodal piano training has causal effects on auditory cortex plasticity. Together with our previous study [21] we conclude that harmonic and rhythmic expectations can be shaped by short-term experience, and that multimodal sensorimotor-auditory training is much more effective than auditory alone training at inducing plastic changes to auditory areas. These results have educational implications in that they show that multimodal training leads to more effective and faster learning than unimodal training.
